# Bilateral Occipital Lobe Herniations Due to Asymmetric Tentorial Defects: Incidental Computed Tomography (CT) Findings & Literature Review

**DOI:** 10.7759/cureus.32117

**Published:** 2022-12-01

**Authors:** Muhammad Shoyab

**Affiliations:** 1 Radiology & Imaging, SHIP International Hospital, Dhaka, BGD

**Keywords:** pediatric neuroimaging, pediatric neurology, developmental defects, occipital herniation, tentorium cerebelli

## Abstract

Congenital defects in the tentorium cerebelli are quite rare occurrences and are often too small and asymptomatic. This is a case report of a female patient aged 11 years, complaining of headache, vertigo, and vomiting. Her computed tomography (CT) images show transtentorial herniation of occipital gyri across a developmental defect involving the anterior free margin of the tentorium cerebelli. Similar cases have been reported in the past as "incidental" and "potentially symptomatic" findings, and in at least one case as a proven pathological findings. Our case is unique in terms of the asymmetric bilateral configuration and comparatively larger size of the defect. We have included a review of the existing medical literature in order to derive learning points for the betterment of our understanding of a rare entity that can have significant implications.

## Introduction

The principal function of the tentorium cerebelli is to prevent direct contact between the cerebral and cerebellar cortices [1]. It also serves to dissipate the weight of the cerebrum towards the occiput instead of directly weighing upon the cerebellum [1]. Focal defect in some part of the tentorium has been attributed to abnormal fusion of the dural reflections during embryonic development [2,3], and have been described as incidental findings in some case reports [2,3]. Most of those reports found no pathophysiological relation between clinical features and imaging findings [4,5]. Our patient may be one of the few [3] where the defect has a larger size than others and may have some correlation with the patient’s symptoms [3].

## Case presentation

Our patient is a girl aged 11 years, complaining of headache, intermittent vertigo, and some episodes of vomiting for approximately a month. The patient is otherwise normal in demeanor as appropriate for her age, with normal involvement at school, games, and social activities. She did not complain of any visual or auditory difficulties and does not need to wear spectacles or hearing aids. Her birth had been without complications, followed by a normal growth pattern. She had received vaccines according to national immunization schedules and is not under any regular medications at present. Bedside neurological examinations were normal, including gait, balance, cranial nerve functions, visual acuity, and visual fields on both sides. As such, computed tomography (CT) was advised to rule out whether her symptoms are being caused by cerebral edema or any underlying optic pathway lesions.

**Figure 1:**
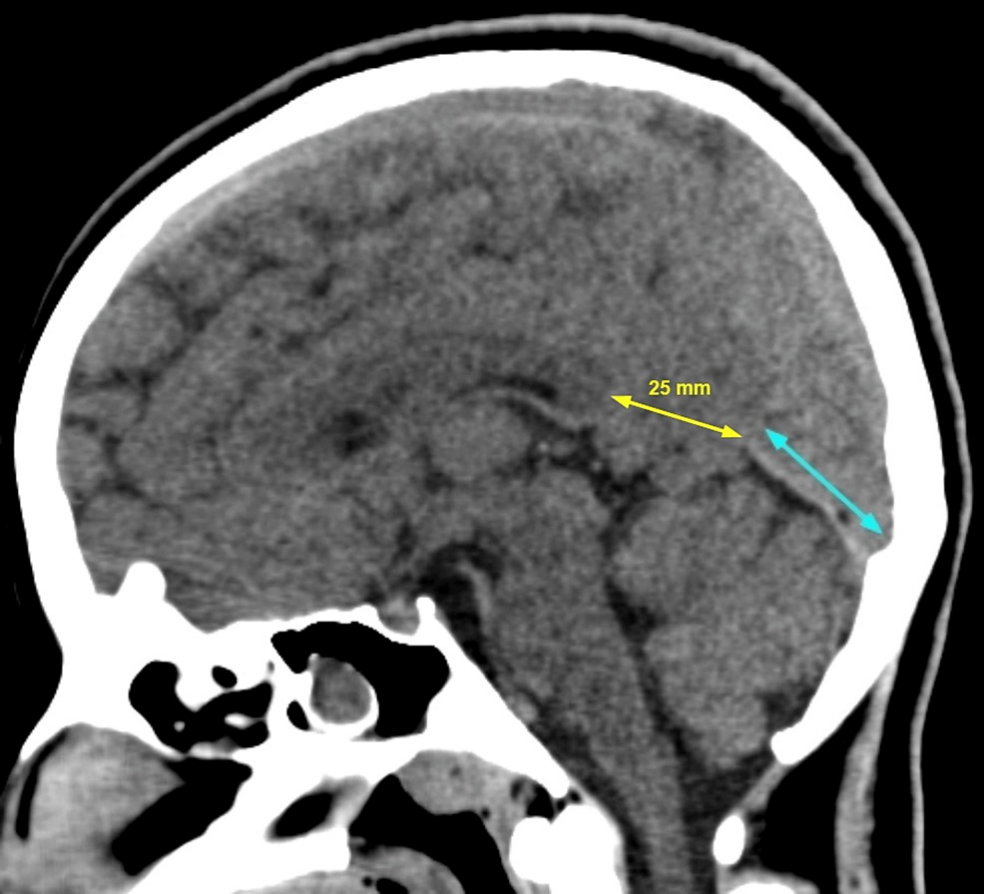
CT, brain window, left paramedian sagittal section. The cyan arrow shows the intact posterior part of the tentorium. Anterior to it is the defect (yellow arrow), allowing herniation of parts of the occipital lobe into the cistern below. CT: computed tomography

**Figure 2:**
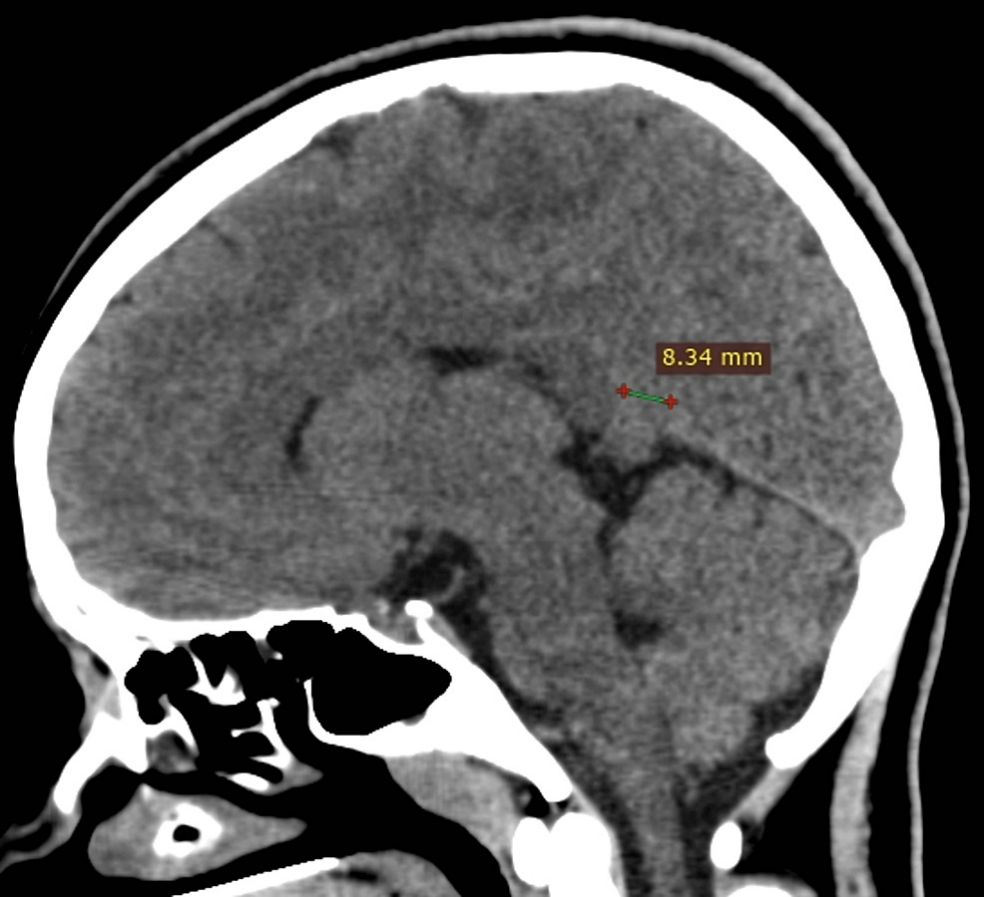
CT, brain window, right paramedian sagittal section. A small (8 mm) defect at the anterior edge of the tentorium, allowing herniation of a small part of the occipital lobe into the cistern below. CT: computed tomography

The CT shows an asymmetric defect involving the anterior free margin of the tentorial notch, spanning both sides of the midline. The defect measures 25 mm on the left side and 8 mm on the right. It allows inferomedial herniation of the precuneus and cuneus of the left occipital lobe (Figure 1), along with the lingual gyrus of the right occipital lobe (Figure 2), into the quadrigeminal cistern below.

**Figure 3:**
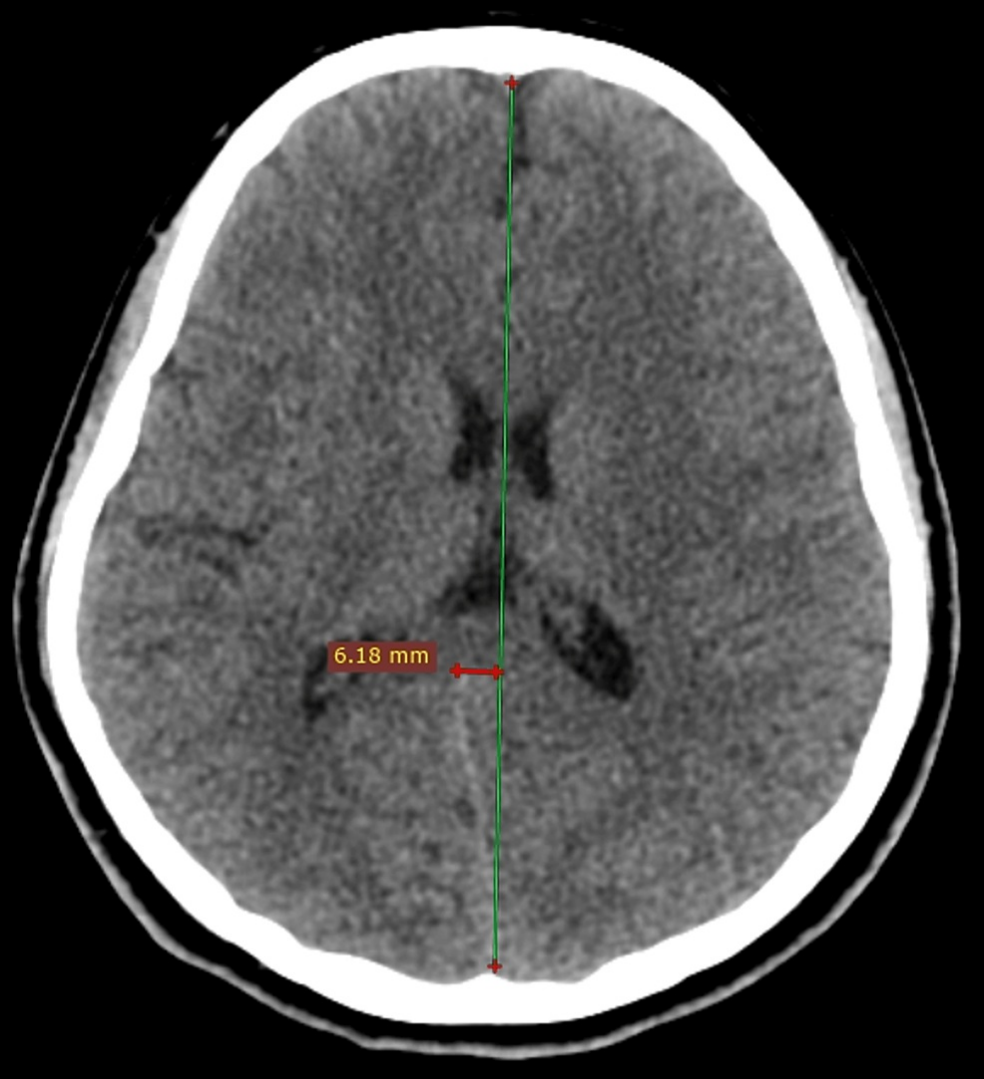
CT, brain window, axial image. The slight rightward shift of posterior falx due to herniation of the occipital lobe through the defect. CT: computed tomography

Also, there is about an 8 mm rightward deviation of the adjacent falx cerebri (Figure 3). Further imaging, such as an MRI of the brain and spine, was not advised on the consideration that the patient did not have any visual field defects or identified mass lesion on the CT. There was no other significant finding on the CT, including no fluid collection in the middle ear or mastoids on either side.

## Discussion

While occipital lobe herniation is mostly considered asymptomatic and unrelated with clinical features, there is at least one case report [3] where the functional anatomy of the herniated portion (medial right occipital gyri) correlated with the patient’s symptoms (left-sided homonymous hemianopia, more prominent in the inferior quadrant). The authors used diffusion tensor imaging (DTI) to verify their interpretation and identified that parts of the optic pathway fibers were indeed trapped within the herniation. Sun et al. [3] commented that occipital herniations may therefore be considered “potentially symptomatic” rather than absolutely “asymptomatic”. They inferred that the effects of such herniations could be related with the size of the defect and the nerves or tracts involved [3].

Herniation occurs across the defect due to the pressure difference, which allows the herniated part to occupy a cistern or to push against another normal structure [5,6]. Such findings on CT or MRI may sometimes mimic a space-occupying lesion or underlying intracranial hypertension [6], which may stimulate further unnecessary imaging or invasive procedure [4-6]. Thus, although most cases of tentorial defect may be free of symptoms, they may not always be free of implications. Therefore, it is important to expand our knowledge regarding these rare congenital or idiopathic abnormalities.

In our patient, the defect has quite a significant size (2.5 cm) on the right side, which allows direct contact between the occipital and cerebellar cortices. However, we could not make a size-by-size comparison with previous case reports, since most of them did not mention any measurements. Sun et al. [3] only stated that their symptomatic patient discussed above had a “moderate-sized” defect on the right side.

In addition to having a defect that is quite large, our patient is also unique in terms of the defect being bilateral and asymmetric, whereas almost all the other case reports have mentioned unilateral findings [2-4,6]. We found only one other report mentioning a bilateral finding [7], but in that case, the defect was rather small and there was no correlated symptom. The authors did not go as far as pointing out whether it was symmetric or asymmetric, quite obviously because they did not consider it necessary as it was not related with the patient’s condition [7]. We feel that there have been other bilateral defects too, which were missed or overlooked by the concerned authors.

Similar to ours, the report by Thomaere et al. [4] describes a patient presenting with vertigo and vomiting, whose MRI revealed herniation of the inferior precuneus. However, the authors considered vertigo to be unrelated with the herniation [4], just like most other case reports. In our view, the lack of correlation is quite due to the broad-spectrum, non-specific, or vague nature of the presenting symptoms [5], accompanied by the so small number of case reports [3] and a general perception that such a focal defect or herniation may be harmless. Such discrepancy may also be attributed to the non-usage of DTI [3] for such cases by most clinicians, radiologists, and researchers alike. Otherwise, we can see that the patients’ presentations follow a common pattern - non-specific features like headache, nausea, and vertigo [3,5] and sometimes raising specific concerns for optic pathway lesions [3]. It may be noted that our patient's CT was also initially advised to rule out cerebral edema or any optic pathway lesion.

It is also interesting to note that the first patient report of a congenital tentorial defect was published only in 1976 [3,8], whereas such defects may have been a part of human embryogenesis for centuries. The reason is very obvious - earlier researchers did not have CT or MRI. This may be compared to our interpretation in a previous paper, where we derived that Tarlov cysts have remained unexplored for decades or centuries until the advent of the 1.5 Tesla MRI in the 1980s [9].

## Conclusions

Focal cortical herniation due to isolated tentorial hypoplasia is most often a benign entity with no complications. However, CT or MRI images thereof may sometimes mimic critical pathologies like space-occupying lesions or underlying intracranial hypertension, since the herniated parenchyma occupies a cistern or pushes against another normal structure. It is thus important to remember in this context that such a herniated portion is essentially normal brain parenchyma and would exhibit a normal appearance on CT and all sequences of MRI, whereas pathological tissue would differ from normal in one sequence or another. Radiologists and clinicians need to remind themselves of this small basic principle, so as to avoid sending the patient for additional imaging or invasive procedures.

Also of importance is the fact that while most herniations are asymptomatic due to very small size, those that are slightly larger may have nerve tracts compressed within the herniated brain matter. This can produce infarct or mass-effect-like symptoms. Therefore, it may be a good practice to make the imaging evaluation complete by means of MR tractography by DTI sequences, which can detect whether any nerve tracts are trapped or compressed. The use of DTI and visual field assessment would be particularly important if a patient presents with relevant symptoms, such as blurred vision, diplopia, vertigo, or vomiting.

## References

[1] Adeeb N, Mortazavi MM, Tubbs RS, Cohen-Gadol AA (2012). The cranial dura mater: a review of its history, embryology, and anatomy. Childs Nerv Syst.

[2] Wahl L, Iwanaga J, Chabot AB, Dumont AS, Tubbs RS (2022). Hypoplasia of the tentorium cerebelli: case report and review of the literature. Kurume Med J.

[3] Sun Y, Bobra S, Kurian C, Ahluwalia-Singh B, Thomas A, Mehta H (2018). MR and diffusion tensor imaging of isolated tentorial hypoplasia. Neurol Clin Pract.

[4] Thomaere E, Schepers S, Termote B, Vanwyck R, Souverijns G (2015). Tentorium hypoplasia with partial occipital lobe herniation. JBR-BTR.

[5] Seok HY, Lee DH (2017). Magnetic resonance imaging of idiopathic herniation of the lingual gyrus: A case report. Investig Magn Reson Imaging.

[6] Agrawal SK, Premi V, Babu CSR (2020). Unilateral tentorial hypoplasia with ipsilateral brain herniation. JMSCR.

[7] Knipe H, Spychalski P (2022). Tentorium hypoplasia with partial occipital lobe herniation. Radiopaedia.org.

[8] Gund A (1976). Hypoplasia of the tentorium with pseudohernia of the occipital lobe into the posterior cranial fossa. Mod Probl Paediatr.

[9] Shoyab M (2021). Tarlov cysts in back pain patients: prevalence, measurement method and reporting points. Br J Radiol.

